# Surgical and Reproductive Outcomes of a Standardized Hysteroscopic Approach to Treat Septate Uterus and Associated Complex Anomalies

**DOI:** 10.3390/jcm15103786

**Published:** 2026-05-14

**Authors:** Ursula Catena, Emma Bonetti Palermo, Federica Pozzati, Federica Bernardini, Giulia Micol Bruni, Federica Campolo, Eleonora La Fera, Michela Zorzi, Angelica Naldini, Francesca Moro, Antonia Carla Testa

**Affiliations:** 1 Dipartimento Scienze della Salute della Donna e del Bambino, Fondazione Policlinico Universitario A. Gemelli, IRCCS, 00168 Rome, Italy; 2 S.C. Ostetricia e Ginecologia, ASST Spedali Civili Brescia, Dipartimento Area Della Donna e Materno Infantile, 25136 Brescia, Italy; 3 Unit of Gynecology and Obstetrics, Department of Women’s and Children’s Health, University of Padova, 35141 Padova, Italy; 4 Unit of Gynecology and Obstetrics, Policlinico Abano Terme, 35031 Abano Terme, Italy; 5 Ovarian Cancer Center, Candiolo Cancer Institute, FPO-IRCCS, 10060 Torino, Italy; 6 G-AURIG—Gemelli Advanced Ultrasound Research and Innovation Group, Fondazione Policlinico Universitario A. Gemelli, IRCCS, 00168 Rome, Italy; 7 UniCamillus International Medical University of Rome, 00131 Rome, Italy; 8 Gemelli Women’s Health Center for Digital and Personalized Medicine, Dipartimento Scienze della Vita e Sanità Pubblica, Università Cattolica del Sacro Cuore, 00168 Rome, Italy

**Keywords:** septate uterus, hysteroscopic metroplasty, mini-resectoscope, congenital uterine anomalies, cervical septum, vaginal septum, duplicate cervix, reproductive outcomes, minimally invasive surgery

## Abstract

**Background/Objectives**: Septate uterus is the most prevalent uterine malformation and is commonly associated with impaired reproductive outcomes. Hysteroscopic metroplasty is the gold standard treatment, but surgical management of complex septate uteri with associated cervical and vaginal anomalies remains challenging. This study aimed to evaluate surgical and reproductive outcomes following a standardized minimally invasive hysteroscopic approach using a 15 Fr bipolar mini-resectoscope across different subtypes. **Methods**: This retrospective single-center, single-surgeon study included women who underwent hysteroscopic correction of partial and complete septate uterus, with or without cervical and/or vaginal anomalies, between January 2021 and January 2025 at the Digital Hysteroscopic Clinic CLASS Hysteroscopy, Fondazione Policlinico A. Gemelli IRCCS, Rome, Italy. Preoperative assessment included three-dimensional transvaginal ultrasound and diagnostic hysteroscopy. All procedures were performed using a standardized hysteroscopic technique with a 15 Fr bipolar mini-resectoscope. Surgical outcomes included operative time and the need for second-step surgery. Reproductive outcomes included clinical pregnancy rate (CPR), live birth rate (LBR), and miscarriage rate (MR). **Results**: A total of 154 patients were included, comprising 70 partial and 84 complete septate uteri; 52.4% of complete septa were associated with cervical and/or vaginal anomalies. Median operative time was 18.0 min for partial septa and 31.0 min for complete septa (*p* < 0.01), and a second surgical step was required in only 5/84 complete septa (5.9%) and in none of the partial septa. Reproductive outcomes were analyzed in a subgroup of 70 patients who attempted conception. After metroplasty, CPR increased from 35.7% to 84.3% (*p* < 0.01), LBR per pregnancy increased from 16.0% to 78.0% (*p* < 0.01), and MR per pregnancy decreased from 84.0% to 10.2% (*p* < 0.01). Postoperative reproductive outcomes appeared comparable between partial and complete septa and according to the presence of associated anomalies. **Conclusions**: A standardized hysteroscopic technique using a 15 Fr bipolar mini-resectoscope is feasible and effective for treating septate uterus, including complex cases associated with cervical and/or vaginal anomalies. Favorable reproductive outcomes can be achieved regardless of anomaly complexity when accurate preoperative diagnosis and a structured surgical approach are applied.

## 1. Introduction

Septate uterus represents the most frequent congenital uterine anomaly, accounting for 80–90% of all malformations of the female genital tract. It is characterized by a normal external uterine profile with an internal fundal indentation that partially or completely divides the uterine cavity [1,2]. This condition is frequently identified during the evaluation of infertility, recurrent pregnancy loss, and adverse obstetrical outcomes [3].

According to the 2013 criteria of the European Society of Human Reproduction and Embryology/European Society for Gynaecological Endoscopy (ESHRE/ESGE), a septate uterus is diagnosed when the internal fundal indentation exceeds 50% of the uterine wall thickness and is classified as partial (U2a) or complete (U2b) [2]. In some cases, uterine septa may be associated with complex cervical and/or vaginal anomalies, such as single cervix with cervical septum (C1), duplicate cervix (C2), and longitudinal non-obstructive vaginal septum (V1), highlighting the need for accurate preoperative assessment and tailored surgical planning [2,4,5,6,7,8].

Three-dimensional transvaginal ultrasound (3D TVUS) is currently considered the reference imaging modality for the diagnosis of uterine anomalies, as it allows accurate assessment of both the uterine cavity and the external uterine contour. Diagnostic hysteroscopy complements 3D TVUS by providing direct visualization of the uterine cavity and enabling more precise characterization of associated lower genital tract anomalies, particularly cervical and vaginal abnormalities. The integration of these diagnostic tools enhances overall diagnostic accuracy and facilitates a comprehensive “one-stop” approach, in which diagnosis and surgical management can be performed within the same setting [9,10,11].

Hysteroscopic metroplasty is considered the gold standard treatment for septate uterus. Among the most recent approaches, a standardized technique using a 15 Fr bipolar mini-resectoscope was described in 2022, offering a minimally invasive and reproducible method for septum resection [12]. However, the surgical management of septate uterus associated with cervical and/or vaginal anomalies remains challenging, particularly in complex malformations where the anatomical configuration requires careful intraoperative assessment and tailored surgical strategies.

The present study aimed to describe the application of a standardized minimally invasive hysteroscopic approach using a 15 Fr mini-resectoscope in women with partial and complete septate uterus, including cases with associated cervical and/or vaginal anomalies, to provide a systematic surgical approach for each anatomical subtype, and to evaluate surgical and reproductive outcomes across different malformation subtypes.

## 2. Materials and Methods

### 2.1. Study Design and Population

This retrospective single-center study was conducted between January 2021 and January 2025 at the Digital Hysteroscopic Clinic CLASS Hysteroscopy, Fondazione Policlinico A. Gemelli IRCCS, Rome, Italy. We included consecutive patients who underwent hysteroscopic correction of partial or complete septate uterus, including patients with associated cervical anomalies (single cervix with cervical septum or duplicate cervix) and/or vaginal anomalies (longitudinal non-obstructive vaginal septum).

Patients with other congenital malformations of the female genital tract, such as bicorporeal uterus, as well as isolated cervical or vaginal septa not associated with uterine septum and obstructive vaginal septum, were excluded.

### 2.2. Ultrasound Evaluation and Diagnostic Hysteroscopy

All patients underwent an integrated preoperative assessment with 3D TVUS and diagnostic hysteroscopy before surgical treatment.

Preoperative 3D TVUS was used to obtain three measurements:

(i) interostial distance (*x*);
(ii) distance from the interostial line to the uterine serosa (*y*);
(iii) distance from the interostial line to the apex of the septum (*z*).

According to the ESHRE/ESGE classification, included malformations were partial (U2a) and complete (U2b) uterine septum, respectively defined as a septum partially dividing the uterine cavity or completely dividing the cavity and reaching the internal cervical os.

Associated cervical anomalies included single cervix with cervical septum (C1) and duplicate cervix (C2), with or without longitudinal non-obstructive vaginal septum (V1).

Diagnostic hysteroscopy was used to confirm the diagnosis of septate uterus, assess the extent of cavity division, and identify associated cervical and/or vaginal anomalies. In particular, hysteroscopic evaluation was used to differentiate cervical septum from duplicate cervix according to the criteria described by Catena et al. [13]. Duplicate cervix was defined as the presence of two distinct external uterine orifices separated by an external cleft and covered by stratified squamous non-keratinized vaginal epithelium. Cervical septum was defined as a single external uterine orifice without an external cleft, with an internal cervical septum covered by endocervical glandular epithelium. In this last case, the cervical anomaly was not in continuity with the vaginal anomaly [13].

### 2.3. Surgical Standardized Technique Using a 15 Fr Bipolar Mini-Resectoscope

All procedures were performed by the same experienced surgeon (U.C.) using a 15 Fr bipolar mini-resectoscope (Karl Storz, Tuttlingen, Germany) and adopting a vaginoscopic approach without cervical dilation under general anesthesia (laryngeal mask).

Normal saline was used as the distension medium. Intrauterine pressure was maintained between 100 and 120 mmHg. Fluid input and output were continuously monitored throughout the procedure. A predefined safety threshold for fluid deficit was set at 2000 mL, and the procedure was interrupted if this limit was approached.

For all types of septa (uterine, cervical and vaginal), incision was performed using a Collins loop electrode, applying a standardized caudo-cranial incision strategy adapted to each malformation subtype.

In some cases of uterine septum, redundant endomyometrial tissue on the anterior and posterior uterine walls was resected using a 90° angled cutting loop [14].

3D TVUS evaluation was performed immediately after surgery.

Pharmacological preoperative preparation of the endometrium was achieved with at least one month of progestin therapy (desogestrel 75 mcg/day or norethisterone acetate 5 mg/day) to reduce endometrial thickness and minimize intraoperative bleeding.

Intraoperative complications (uterine perforation, fluid overload and bleeding requiring intervention) and postoperative complications (infection and intrauterine adhesions at follow-up hysteroscopy) were recorded.

The standardized surgical steps according to each anatomical variant are reported below.

#### 2.3.1. Partial and Complete Septate Uterus (U2aC0V0 and U2bC0V0)

In isolated partial (U2a) or complete (U2b) septate uterus, the septum was incised along the midline in a caudo-cranial direction, starting from the septum apex and proceeding toward the interostial plane.

**Figure 1:**
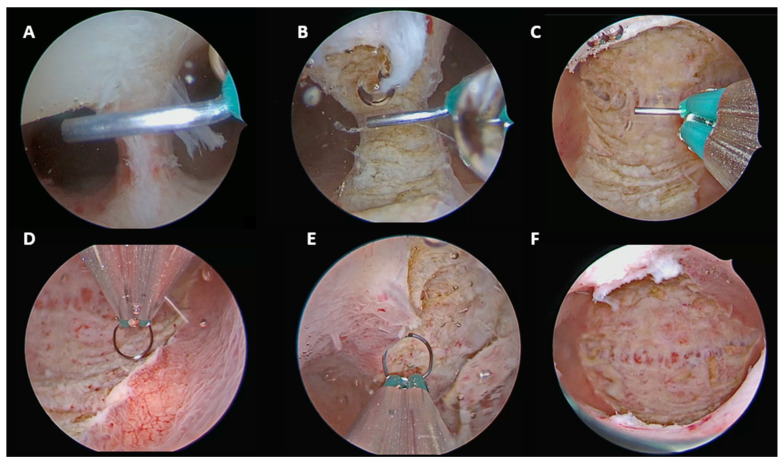
U2aC0V0 and U2bC0V0. (**A**) Initial incision from the septal apex. (**B**) Mid-septal incision in a caudo-cranial direction. (**C**) Completion of septum incision up to the interostial plane. (**D**) Resection of redundant endomyometrial tissue from the posterior wall. (**E**) Resection of redundant endomyometrial tissue from the anterior wall. (**F**) Final panoramic view of the uterine cavity.

The tubal ostia were used as constant anatomical landmarks. The procedure was considered complete when both ostia were clearly visible in a panoramic view and the instrument could move freely between them, confirming the presence of a single unified cavity (Figure 1).

Final anatomical classification according to the ESHRE/ESGE system: U0C0V0.

#### 2.3.2. Complete Septate Uterus with Single Cervix with Cervical Septum and Longitudinal Non-Obstructive Vaginal Septum (U2bC1V1)

**Figure 2:**
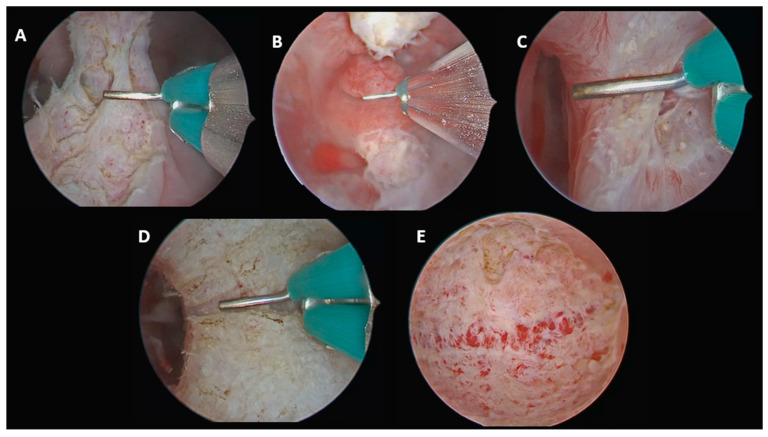
U2bC1V1. (**A**) Incision of the vaginal septum. (**B**) Visualization of the single cervix with cervical septum after vaginal septum incision. (**C**) Caudo-cranial incision of the cervical septum continuing into the uterine septum. (**D**) Incision of the uterine septum. (**E**) Final panoramic view of the uterine cavity.

In this case, the procedure was performed stepwise. First, the longitudinal non-obstructive vaginal septum was fully incised under direct vaginoscopic vision to allow complete access to the cervical region. The cervical septum was then divided using the same midline caudo-cranial incision, followed by incision of the uterine septum up to the interostial plane. This continuous approach restored a single uterine cavity, a single cervix and a normal vaginal canal (Figure 2).

Final anatomical classification according to the ESHRE/ESGE system: U0C0V0.

#### 2.3.3. Complete Septate Uterus with Duplicate Cervix and Longitudinal Non-Obstructive Vaginal Septum (U2bC2V1), Without Communication Between the Two Uterine Hemicavities

**Figure 3:**
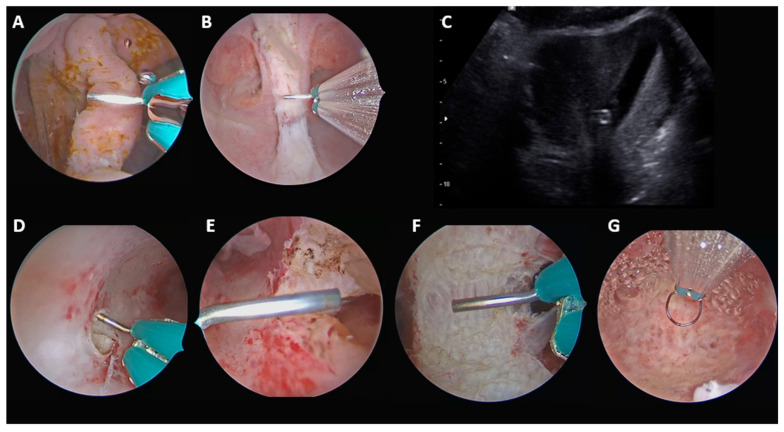
U2bC2V1, without communication between the two hemiuteri. (**A**) Beginning of vaginal septum incision. (**B**) Completion of vaginal septum incision up to the level of the two cervices. (**C**) Real-time ultrasound view guiding the supracervical incision. (**D**) Supracervical incision to create communication between the two uterine hemicavities under transabdominal ultrasound guidance. (**E**) Creation of communication between the two uterine hemicavities, allowing subsequent uterine septum incision. (**F**) Incision of the uterine septum. (**G**) Final panoramic view of the uterine cavity.

In this setting, the procedure was also performed stepwise. The vaginal septum was incised first, allowing clear access to both cervices. Under real-time transabdominal (or transrectal, in case of obese patients or retroverted uterus) ultrasound guidance, the uterine septum was then incised at the thinnest isthmic–supracervical point to create a passage between the two uterine hemicavities. Once communication had been established, the uterine septum was incised up to the interostial plane, with preservation of both cervices (Figure 3).

Final anatomical classification according to the ESHRE/ESGE system: U0C2V0.

#### 2.3.4. Complete Septate Uterus with Duplicate Cervix and Longitudinal Non-Obstructive Vaginal Septum (U2bC2V1), with Communication Between the Two Uterine Hemicavities

**Figure 4:**
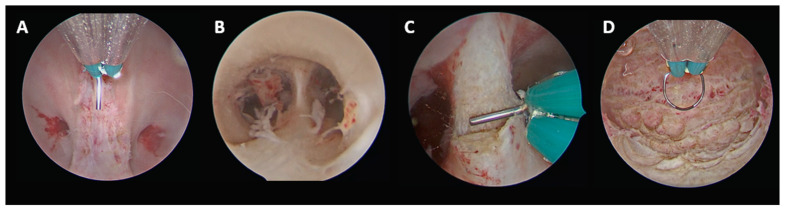
U2bC2V1, with communication between the two uterine hemicavities. (**A**) Vaginal septum incision up to the level of the two cervices. (**B**) Hysteroscopic view showing the uterine septum and the existing communication between the two uterine hemicavities. (**C**) Beginning of uterine septum incision along the midline. (**D**) Final panoramic view of the uterine cavity.

When communication between the two uterine hemicavities was already present, the procedure was technically less complex. As described for the previous subgroup, the vaginal septum was incised first to allow access to both cervices. The uterine septum was then divided along the midline up to the interostial plane using the same caudo-cranial approach guided by the tubal ostia. The pre-existing communication facilitated unification of the two hemicavities without the need for real-time ultrasound guidance (Figure 4).

Final anatomical classification according to the ESHRE/ESGE system: U0C2V0.

#### 2.3.5. Complete Septate Uterus with Duplicate Cervix and Normal Vagina (U2bC2V0), Without Communication Between the Two Uterine Hemicavities

As described for patients with U2bC2V1 without communication between the two uterine hemicavities, real-time transabdominal ultrasound guidance was used to create a passage between the two uterine hemicavities at the isthmic–supracervical plane, where the septum was thinner. In the absence of a vaginal septum, the procedure directly started at the uterine cavity level. Once communication had been established, the uterine septum was completely resected up to the interostial plane using the standard caudo-cranial approach, resulting in a single unified uterine cavity while preserving both cervices intact.

Final anatomical classification according to the ESHRE/ESGE system: U0C2V0.

#### 2.3.6. Complete Septate Uterus with Single Cervix and Cervical Septum, with Normal Vagina (U2bC1V0)

As described for U2bC1V1, the cervix was first inspected to confirm the presence of a single cervix with central septation. In the absence of a vaginal septum, the procedure directly started with incision of the cervical septum in a caudo-cranial direction. The incision was then continued cranially into the uterine septum up to the interostial line, following the same anatomical landmarks described above.

Final anatomical classification according to the ESHRE/ESGE system: U0C0V0.

### 2.4. Postoperative Office Evaluation

All patients underwent postoperative office evaluation 30–40 days after the surgical procedure. This step included 3D TVUS and office hysteroscopy, during which additional refinement of the uterine cavity (through fundal cuts with 5 Fr scissors) was performed, if needed.

Refinement was defined as minor fundal cuts performed at the site of mild myometrial remodeling, aimed at improving the regularity of the uterine cavity, and carried out during follow-up hysteroscopy in an outpatient setting. By contrast, second-step surgery was defined as an additional operative procedure under general anesthesia required to complete septum resection in cases of residual septum.

### 2.5. Reproductive Outcomes

Reproductive outcomes were assessed after the postoperative hysteroscopic office control performed 30–40 days after the surgical procedure. Only patients who attempted conception after metroplasty were included in the reproductive outcome analysis. Patients who did not attempt conception during the follow-up period, including those undergoing pre-conceptional correction or with delayed or not yet initiated attempts at conception, were excluded.

The clinical pregnancy rate (CPR), live birth rate (LBR), and miscarriage rate (MR) were evaluated both before and after hysteroscopic metroplasty. CPR was defined as any pregnancy confirmed by ultrasound with visualization of a gestational sac and fetal heartbeat. LBR was defined as the delivery of a live infant beyond 24 weeks of gestation. MR was defined as the proportion of clinical pregnancies ending before 24 weeks of gestation. CPR was calculated as the proportion of women (with ≥1 clinical pregnancy), whereas LBR and MR were calculated per clinical pregnancy, considering the outcome of the first clinical pregnancy recorded for each patient. Mode of conception (spontaneous vs. assisted reproductive technology, ART), mode of delivery (vaginal delivery or cesarean section), and obstetric complications were also recorded.

### 2.6. Statistical Analysis

Categorical variables were expressed as absolute numbers and percentages. Continuous variables were reported as medians and interquartile ranges (IQRs).

Comparisons between categorical variables were performed using the Chi-square test or Fisher’s exact test, as appropriate, while continuous variables were compared using the Mann–Whitney U test.

Comparisons of outcomes before and after treatment were analyzed using McNemar’s test for paired outcomes, while comparisons between independent proportions were performed using the Chi-square test or Fisher’s exact test, as appropriate.

Statistical analyses were conducted using IBM SPSS Statistics software (version 26; IBM Corp., Armonk, NY, USA), and a *p*-value < 0.05 was considered statistically significant for all tests.

### 2.7. Ethical Approval

The study was conducted in accordance with the principles of the Declaration of Helsinki. Ethical approval was obtained from the regional Medical Ethics Review Committee (Comitato Etico Territoriale Lazio Area 3) under reference number ID 6716 (approval date: 17 September 2024). Written informed consent was obtained from all participants.

## 3. Results

### 3.1. Population Characteristics

A total of 154 patients were included in the study, comprising 70 women with partial uterine septum (45.4%) and 84 with complete uterine septum (54.6%).

Table 1 summarizes the anatomical subtypes of uterine septum and the associated cervical and/or vaginal anomalies. Among the 84 patients with complete uterine septum, 40 (47.6%) had an isolated uterine septum, while more than half (44, 52.4%) presented with associated cervical and/or vaginal anomalies. Specifically, seventeen patients (20.2%) had an associated cervical septum, one (1.2%) had both cervical and vaginal septum, one (1.2%) had double cervix without communication between the two uterine hemicavities, nineteen (22.6%) had duplicate cervix with a non-obstructive vaginal septum and no communication between the two uterine hemicavities, and six (7.2%) had duplicate cervix with non-obstructive vaginal septum and communication between the two uterine hemicavities. Among the seventeen cases with both uterine and cervical septum, only one had no continuity between the two anomalies. In the single case with associated uterine, cervical and vaginal septa, no continuity between the cervical and vaginal anomalies was observed.

Baseline characteristics of the study population, stratified by partial and complete septate uterus, are reported in Table 2.

The median age at surgery in the overall population was 32 years (IQR 28–35). Although not statistically significant, patients with partial uterine septum were older than those with complete septate uterus (33 years (IQR 28–36) vs. 31 years (IQR 28–35), *p* = 0.15). No significant between-group differences were observed for body mass index (22.5 kg/m^2^ (IQR 20.3–25.0) vs. 21.9 kg/m^2^ (IQR 20.5–23.8), *p* = 0.36), previous ectopic pregnancy (1.4% vs. 1.2%, *p* = 1.00), or concomitant presence of fibroids (20.0% vs. 19.0%, *p* = 0.88).

Implantation failure after ART (assisted reproductive technology) was significantly more frequent in the partial septum group than in the complete septum group (6/70, 8.6% vs. 1/84, 1.2%, *p* = 0.04). A history of miscarriage was also significantly more common among women with partial uterine septum (23/70, 32.9% vs. 9/84, 10.7%, *p* < 0.01), particularly in patients with a history of one miscarriage only (15/70, 21.4% vs. 3/84, 3.6%, *p* < 0.01).

Regarding previous obstetrical history, only two patients with partial uterine septum had a previous term vaginal delivery. Among patients with complete septate uterus, five had previous live births: two term cesarean deliveries in cases of isolated complete uterine septum and three preterm cesarean deliveries, including two in isolated complete septate uterus and one in a U2bC2V1 case.

### 3.2. Ultrasound Findings

The preoperative 3D TVUS measurements are summarized in Table 3.

The median interostial distance (*x*) was 32.0 mm (IQR 27.0–39.0) in patients with partial uterine septum and 34.0 mm (IQR 29.0–41.0) in those with complete uterine septum, with no significant difference between groups (*p* = 0.09). Similarly, the distance from the interostial line to the serosa (*y*) did not differ significantly between the two groups (7.8 mm (IQR 6.0–9.0) vs. 7.0 mm (IQR 5.8–9.0), *p* = 0.30). In contrast, septal depth (*z*) was significantly superior in patients with complete uterine septum than in those with partial uterine septum (30.0 mm (IQR 26.0–36.0) vs. 14.3 mm (IQR 11.7–18.2), *p* < 0.01).

### 3.3. Surgical Features

Table 4 summarizes the surgical characteristics of the patients included in the study, stratified according to the type of septum (partial vs. complete).

Preoperative progestin therapy was used in 61/70 patients (87.1%) with partial septate uterus and in 80/84 (95.2%) with complete septate uterus, without a significant difference between groups (*p* = 0.09).

At the completion of septum incision, resection of redundant endomyometrial tissue from the anterior and posterior uterine walls was performed in almost all patients with complete septate uterus (83/84, 98.8%), compared with 48/70 (68.6%) in the partial septum group (*p* < 0.01).

Median surgical time was significantly longer for complete septa compared with partial septa (31.0 min (IQR 22.0–45.0) vs. 18.0 min (IQR 14.0–24.0), *p* < 0.01).

No intraoperative or postoperative complications were observed across all groups.

A second surgical step was not required in any patient with partial septate uterus, whereas 5 out of 84 patients with complete septate uterus (5.9%) required an additional procedure (*p* = 0.04). Specifically, one case of isolated U2b required re-intervention due to residual septum detected at follow-up; one U2bC1 case was interrupted due to rising risk of fluid overload during concomitant myomectomy; and three U2bC2V1 cases required a second surgical step, two due to prolonged operative time and one due to suboptimal ultrasound visualization related to marked uterine retroversion.

No significant differences were observed between groups in fundal thickness at the end of the procedure (10.8 mm (IQR 10.1–11.9) vs. 10.5 mm (IQR 10.0–11.2), *p* = 0.35) or at the postoperative office control (10.5 mm (IQR 10.0–11.0) vs. 10.2 mm (IQR 10.0–11.1), *p* = 0.21). Postoperative evaluation was performed after a median interval of 40 days (IQR 35–55) in the partial septum group and 41 days (IQR 35–51) in the complete septum group (*p* = 0.78). Likewise, the need for cavity refinement at postoperative office control was comparable between groups (42/70, 60.0% vs. 58/84, 69.0%, *p* = 0.24).

### 3.4. Reproductive Outcomes

Reproductive outcomes were analyzed in a subgroup of 70 patients who attempted conception after metroplasty, including 38 with partial septate uterus and 32 with complete septate uterus (Table 5). Among patients with complete septa, 16 had isolated uterine septum and 16 had associated cervical and/or vaginal anomalies. Assisted reproductive technology (ART) was used in 6/38 (15.8%) patients with partial septum and 4/32 (12.5%) with complete septum (*p* = 0.75).

An overall improvement in reproductive outcomes was observed after hysteroscopic metroplasty. The clinical pregnancy rate (CPR) increased from 35.7% to 84.3% (*p* < 0.01). The live birth rate (LBR) per pregnancy increased from 16.0% to 78.0% (*p* < 0.01), whereas the miscarriage rate (MR) per pregnancy decreased from 84.0% to 10.2% (*p* < 0.01).

Among patients with partial septate uterus, significant improvements were observed across all reproductive outcomes. CPR increased from 44.7% to 84.2% (*p* < 0.01), LBR per pregnancy increased from 11.8% to 84.4% (*p* < 0.01), and MR decreased from 88.2% to 3.1% (*p* < 0.01).

Among patients with complete septate uterus, improvements were also observed. In patients with isolated complete septum, CPR increased from 18.7% to 81.2% (*p* < 0.01), LBR increased from 33.3% to 69.2% (*p* = 0.03), and MR decreased from 66.6% to 15.4%, although this reduction did not reach statistical significance (*p* = 0.09). In patients with complete septum and associated cervical or vaginal anomalies, CPR increased from 31.2% to 87.5% (*p* < 0.01), LBR increased from 20.0% to 71.4% (*p* = 0.04), and MR decreased from 80.0% to 21.4% (*p* = 0.02).

The median follow-up duration was 28 months (IQR 20–38). At the time of follow-up closure, seven pregnancies were ongoing (7/59, 11.9%), including four in the partial septum group (4/32, 12.5%), two in the isolated complete septum group (2/13, 15.4%), and one in the complete septum group with associated cervical or vaginal anomalies (1/14, 7.1%).

As shown in Table 6, postoperative reproductive outcomes appeared comparable between partial and complete septate uterus, as well as between complete septa with and without associated cervical or vaginal anomalies.

Regarding mode of delivery, among patients with partial septa who achieved a live birth (n = 27), eighteen delivered vaginally and nine by cesarean section. In the group with complete septa without associated cervical or vaginal anomalies (n = 9), five patients had a vaginal delivery and four underwent cesarean section. Among patients with complete septate uterus and associated cervical and/or vaginal anomalies (n = 10), eight deliveries were by cesarean section (three U2bC1V0 and five U2bC2V1), whereas the remaining two delivered vaginally (one U2bC1V1 and one U2bC2V1).

Regarding obstetrical complications, two cases of postpartum hemorrhage, two cases of retained products of conception (RPOC), and two preterm deliveries (due to chorioamnionitis and preeclampsia) were observed.

## 4. Discussion

This single-center, single-surgeon study supports the feasibility, safety and standardized application of a hysteroscopic technique using a 15 Fr bipolar mini-resectoscope for the correction of both partial and complete septate uterus, including cases with associated cervical and/or vaginal anomalies. Notably, more than half of the treated septa were complete, and more than half of these were associated with additional cervical and/or vaginal abnormalities, highlighting the complexity and heterogeneity of the population affected by these genital tract anomalies.

In our series, partial septa were more frequently associated with a history of implantation failure after ART and miscarriage than complete septa, with failed implantation after embryo transfer reported in 8.6% of partial septa compared with 1.2% of complete septa (*p* = 0.04), and miscarriage occurring in 32.9% versus 10.7%, respectively (*p* < 0.01). These findings suggest that partial septa may remain clinically unrecognized for longer and may more often be diagnosed after implantation failure or early pregnancy loss rather than through earlier anatomical diagnosis. Consistent with this interpretation, patients with partial septa were slightly older at the time of surgery than those with complete septa (median 33 vs. 31 years, *p* = 0.15), although this difference did not reach statistical significance.

By contrast, complete septa in our cohort appeared more frequently associated with adverse obstetrical outcomes, particularly preterm delivery. Although the number of previous deliveries was limited, all preterm births recorded in the study population occurred in patients with complete septate uterus, accounting for three of the five deliveries observed in this group. This may reflect the different clinical expression of the two subtypes: partial septa may be more often linked to subfertility and miscarriage, whereas complete septa may be often linked to adverse obstetrical outcomes, particularly preterm birth. Moreover, complete septa are generally easier to recognize on imaging and are therefore more frequently diagnosed during pre-conceptional assessment, potentially leading to earlier surgical correction.

These findings support the importance of systematic diagnosis and appropriate management in women affected by genital tract anomalies who wish to conceive. Although the indications for hysteroscopic metroplasty remain debated in the literature and should be individualized according to the clinical setting, as also reflected in the 2024 ASRM guideline [15], our cohort included a high proportion of patients with severe anatomical distortion and complex malformations. In this context, our findings also align with the concept of a proactive surgical approach, as suggested by the systematic review and meta-analysis by Noventa et al., which reported improved reproductive outcomes after hysteroscopic metroplasty [3].

All patients in our series underwent a standardized preoperative diagnostic work-up combining 3D TVUS and diagnostic hysteroscopy. This integrated assessment was essential to differentiate partial from complete septa and to identify any associated cervical and/or vaginal anomalies, allowing accurate surgical planning according to the specific anatomical subtype. In our cohort, no patient required magnetic resonance imaging, confirming that the combined use of 3D TVUS and hysteroscopy provided sufficient anatomical detail for both diagnosis and surgical management plan in all cases.

On 3D ultrasound reconstruction, the median septal depth was twice as great in complete septa compared to partial ones (30.0 mm vs. 14.3 mm, *p* < 0.01), confirming the relevance of this measurement for characterizing anomaly severity. Interestingly, the interostial distance tended to increase with septal depth (32 mm partial vs. 34 mm complete, *p* = 0.09), while the fundal myometrial thickness remained comparable between groups (7.8 mm partial vs. 7.0 mm complete, *p* = 0.30), supporting the concept that a deeper septum modifies cavity geometry without significantly affecting the fundal myometrial wall.

The standardized hysteroscopic technique used in this study employed a 15 Fr bipolar mini-resectoscope with a Collins loop electrode for the treatment of uterine, cervical and vaginal septa. For uterine septa, the procedure followed the standardized approach described by Catena and was based on a midline caudo-cranial incision performed under direct hysteroscopic visualization [12]. The technique relied on constant surgical principles, including identification of the tubal ostia as anatomical landmarks, progressive septal incision, and preservation of a residual fundal myometrial thickness of 10–15 mm; when needed, resection of redundant endomyometrial tissue was performed [16,17].

The use of the 15 Fr bipolar mini-resectoscope represented a technical advantage. Its reduced caliber allowed atraumatic access with a vaginoscopic approach, avoiding cervical dilation even in anatomically complex cases with limited intracavitary space [18]. This is particularly relevant in septate uterus also involving the cervix, in which the presence of the septum itself reduces the working space. In this setting, the mini-resectoscope provided good maneuverability and precise control of the incision, while allowing effective bipolar cutting and coagulation.

Endometrial preparation with progestin therapy may be considered to reduce endometrial thickness and minimize intraoperative bleeding [19]. In our cohort, preoperative progestin therapy was used in most patients, without significant differences between partial and complete septa (87.1% vs. 95.2%, *p* = 0.09). The small proportion of patients who did not receive preoperative progestin therapy was mainly related to individual clinical factors, patient preference or compliance, and logistical reasons (e.g., timing of surgery). Removal of redundant endomyometrial tissue was significantly more frequent in complete than in partial septa (98.8% vs. 68.6%, *p* < 0.01), supporting its particular relevance in longer septa for restoring a more physiological cavity morphology. In addition, tissue removal allows histological evaluation, which may represent a further advantage for investigating concomitant associated pathologies [14].

Throughout the series, fundal myometrial thickness remained stable both immediately after surgery and at one-month follow-up, regardless of septum type. Similarly, the need for minor fundal refinement at one-month follow-up did not differ significantly between partial and complete septum groups (60.0% vs. 69.0%, *p* = 0.24). This finding supports the concept that uterine remodeling after hysteroscopic metroplasty is a physiological process related to the intrinsic histological features of the septum and to the myometrium’s ability to reshape and adapt after surgery, and does not depend on septum extent when the septum has been completely removed. Such refinement was safely performed with miniaturized 5 Fr scissors in an outpatient setting.

Conversely, the need for a second surgical step to complete septum resection was not observed in partial septa, whereas 5 out of 84 patients with complete septa (5.9%) required an additional procedure (*p* = 0.04). In our experience, these cases were mainly related to intraoperative interruption due to an increased risk of fluid overload during concomitant procedures (e.g., myomectomy) or occurred in technically demanding U2bC2V1 anomalies, particularly in the presence of marked uterine retroversion. In these situations, the procedure was completed in a second step to ensure safety. Overall, this suggests that the need for a second surgical step is primarily related to the intrinsic complexity of specific anatomical variants within our cohort.

Regarding the complex anomalies associated with complete septum, duplicate cervix with a longitudinal non-obstructive vaginal septum (U2bC2V1) represented the most frequent complex variant in our series, accounting for 25 out of 84 complete septa. In nineteen of these, the two uterine hemicavities were non-communicating, requiring the creation of a passage to unify the cavities while preserving the cervical duplication. In our experience, this delicate step was performed using a real-time transabdominal ultrasound-guided approach combined with the use of a 15 Fr mini-resectoscope. This method allows a controlled incision at the isthmic–supracervical plane, where the septum is thinner, ensuring safe communication between the two uterine hemicavities and reducing the risk of false pathways. This approach has been recently detailed in a dedicated series by Catena et al., including 28 consecutive U2bC2V1 cases treated between January 2021 and May 2025, of which 22 presented without communication between the two uterine hemicavities, showing a progressive reduction in operative time across consecutive procedures and suggesting a learning curve [20]. Compared to other techniques described in the literature, such as placement of a Hegar dilator as a guiding landmark or the use of a Foley catheter balloon in the contralateral hemicavity, our method avoids blind mechanical maneuvers and minimizes trauma by relying entirely on direct hysteroscopic incision under continuous ultrasound guidance [21,22]. Notably, one patient with a preoperative U2bC2V1 anomaly, corrected to U0C2V0 after surgery, subsequently achieved a vaginal delivery managed at our tertiary referral center.

A clear distinction between a single cervix with cervical septum (C1) and a duplicate cervix (C2) is essential, as it guides the surgical plan. As detailed by Catena et al., a duplicate cervix shows two separate external cervical os with an intercervical cleft covered by stratified squamous non-keratinized vaginal epithelium, while a cervical septum appears as a single external uterine orifice without an external cleft, with an internal cervical septum covered by endocervical glandular epithelium [13]. This difference is crucial because a duplicate cervix should be preserved, whereas a single cervix with cervical septum can be safely incised during metroplasty [6,16,23].

In our cohort, reproductive outcomes after hysteroscopic metroplasty were favorable in the overall population, with CPR increasing from 35.7% to 84.3% (*p* < 0.01), LBR per pregnancy from 16.0% to 78.0% (*p* < 0.01), and MR per pregnancy decreasing from 84.0% to 10.2% (*p* < 0.01). This favorable trend was observed across all subgroups of septate uterus, including complex cases, with CPR exceeding 80% in all categories. Overall, these findings suggest that a standardized technique using a mini-resectoscope can be applied across a wide spectrum of genital tract anomalies, from isolated partial septa to the most complex complete septa with cervical and/or vaginal extensions.

Importantly, postoperative reproductive outcomes appeared comparable between partial and complete septate uterus, as well as between complete septa with and without associated cervical or vaginal anomalies, suggesting that, when metroplasty is performed using a standardized and anatomically tailored approach, postoperative reproductive outcomes may become comparable regardless of anomaly complexity. However, these analyses were based on relatively small numbers; therefore, these findings should be interpreted as descriptive and hypothesis-generating.

A recent multicenter retrospective series by Zizolfi et al., including 101 patients, reported surgical and reproductive outcomes after hysteroscopic metroplasty in women with complete septate uterus with or without associated cervical and/or vaginal anomalies [24]. In that study, reproductive outcomes were available in 66 patients, and no significant differences in CPR, LBR, and MR were observed according to the presence of associated cervical or vaginal anomalies. A second surgical resection for residual septum was required in 57 cases (56%), without differences between groups. However, that study reported the use of different surgical techniques, including 5 Fr electrodes, 15 Fr bipolar mini-resectoscopes, and 27 Fr resectoscopes, reflecting a more heterogeneous operative setting.

The main strengths of our study include the systematic use of an integrated preoperative diagnostic work-up and the application of a uniform standardized hysteroscopic technique across the full spectrum of genital tract anomalies, including the most complex forms, all performed by a single experienced surgeon, thereby eliminating inter-operator variability.

Limitations include its retrospective and single-center design without a control group, the relatively small number of patients in some subgroups, and the analysis of reproductive outcomes only in patients attempting conception after surgery, which may limit external generalizability. Additionally, the presence of ongoing pregnancies at the time of data closure may affect final reproductive outcomes with longer follow-up. However, the detailed anatomical characterization of the cohort and the reproducibility of the surgical method provide a practical reference for referral centers aiming to implement a similar standardized approach.

Future studies should focus on longer-term reproductive and obstetrical outcomes, including pregnancy course and delivery management, particularly in women with persistent cervical anomalies. Prospective multicenter studies comparing this standardized mini-resectoscopic approach with other operative strategies may help to further define its role and support broader adoption in reproductive surgery.

## 5. Conclusions

This study supports the feasibility, safety, and standardized application within a single expert center of a hysteroscopic approach using a 15 Fr bipolar mini-resectoscope for the treatment of septate uterus, including complex cases with cervical and/or vaginal anomalies. With systematic preoperative assessment using 3D TVUS and diagnostic hysteroscopy, this approach allowed effective correction across different anatomical subtypes. After metroplasty, improved reproductive outcomes were observed, with similar outcomes regardless of anomaly complexity. These findings highlight the importance of accurate diagnosis, standardized surgical technique, and referral center expertise. Further prospective multicenter studies with longer follow-up are warranted.

## Figures and Tables

**Table 1:** Subtypes of septate uteri and associated cervical and vaginal anomalies.

Septate Uteri (n = 154)		Complete Septate Uteri (n = 84)	
Partial (U2a)	70 (45.4%)		
Complete (U2b)	84 (54.6%)		
		Without cervical or vaginal anomalies (U2bC0V0)	40 (47.6%)
		With cervical septum * (U2bC1V0)	17 (20.2%)
		With cervical septum and non-obstructive vaginal septum ** (U2bC1V1)	1 (1.2%)
		With duplicate cervix (U2bC2V0) without communication between uterine hemicavities	1 (1.2%)
		With duplicate cervix and vaginal non-obstructive septum (U2bC2V1) without communication between uterine hemicavities	19 (22.6%)
		With duplicate cervix and vaginal non-obstructive septum (U2bC2V1) with communication between uterine hemicavities	6 (7.2%)

* Among the seventeen cases, one had no continuity between the uterine and cervical anomalies; ** In the single case with uterine, cervical, and vaginal septum, there was no continuity between the cervical and vaginal anomaly.

**Table 2:** Preoperative characteristics of the population, divided into partial and complete septa.

	Population (n = 154)	Partial Septum (n = 70)	Complete Septum (n = 84)	*p*
Age (years)	32 (28–35)	33 (28–36)	31 (28–35)	0.15
BMI (kg/m^2^)	22.2 (20.4–24.3)	22.5 (20.3–25.0)	21.9 (20.5–23.8)	0.36
EP	2 (1.3%)	1 (1.4%)	1 (1.2%)	1.00
Implantation failure after ET	7 (4.5%)	6 (8.6%)	1 (1.2%)	0.04
Presence of fibroids	30 (19.5%)	14 (20.0%)	16 (19.0%)	0.88
Miscarriage rate	32 (20.8%)	23 (32.9%)	9 (10.7%)	<0.01
1 miscarriage	18 (11.7%)	15 (21.4%)	3 (3.6%)	<0.01
>1 miscarriage	14 (9.1%)	8 (11.4%)	6 (7.1%)	0.36
Live births	7 (4.5%)	2 (2.9%) *	5 (6.0%) **	0.46

BMI = body mass index; EP = ectopic pregnancy; ET = embryo transfer. * Two term vaginal deliveries; ** Two term cesarean sections and three preterm cesarean sections.

**Table 3:** Preoperative 3D transvaginal ultrasound measurements of partial and complete septa.

	Partial Septum (n = 70)	Complete Septum (n = 84)	*p*
*x* (mm)	32.0 (27.0–39.0)	34.0 (29.0–41.0)	0.09
*y* (mm)	7.8 (6.0–9.0)	7.0 (5.8–9.0)	0.30
*z* (mm)	14.3 (11.7–18.2)	30.0 (26.0–36.0)	<0.01

*x*: interostial distance; *y*: distance between the interostial line and the serosa; *z*: the depth of the septum.

**Table 4:** Surgical features of partial and complete septa.

	Partial Septum (n = 70)	Complete Septum (n = 84)	*p*
Preoperative progestin therapy	61 (87.1%)	80 (95.2%)	0.09
Endo-myometrium resection	48 (68.6%)	83 (98.8%)	<0.01
Surgical time (minutes)	18.0 (14.0–24.0)	31.0 (22.0–45.0)	<0.01
Fundal thickness (mm) after surgery	10.8 (10.1–11.9)	10.5 (10.0–11.2)	0.35
Need for second-step surgery	0 (0.0%)	5 (5.9%)	0.04
Time until postoperative control (days)	40 (35–55)	41 (35–51)	0.78
Need for refinement at postoperative control	42 (60.0%)	58 (69.0%)	0.24
Fundal thickness at postoperative control (mm)	10.5 (10.0–11.0)	10.2 (10.0–11.1)	0.21

**Table 5:** Comparison of reproductive outcomes before and after metroplasty in all populations, in partial septum, and in complete septum with and without cervical/vaginal anomalies.

	Population (n = 70)			Partial Septum (n = 38)			Complete Septum Without Cervical/Vaginal Anomalies (n = 16)			Complete Septum with Cervical/Vaginal Anomalies (n = 16)		
	Before	After	*p*	Before	After	*p*	Before	After	*p*	Before	After	*p*
CPR	25/70; 35.7% (25.5–47.4)	59/70; 84.3% (74.0–91.0)	<0.01	17/38; 44.7% (30.1–60.3)	32/38; 84.2% (69.6–92.6)	<0.01	3/16; 18.7% (6.6–43.0)	13/16; 81.2% (57.0–93.4)	<0.01	5/16; 31.2% (14.2–55.6)	14/16; 87.5% (64.0–96.5)	<0.01
LBR	4/25; 16.0% (6.4–34.7)	46/59; 78.0% (65.9–86.6)	<0.01	2/17; 11.8% (3.3–34.3)	27/32; 84.4% (68.2–93.1)	<0.01	1/3; 33.3% (6.1–79.2)	9/13; 69.2% (42.4–87.3)	0.03	1/5; 20.0% (3.6–62.4)	10/14; 71.4% (45.4–88.3)	0.04
MR	21/25; 84.0% (65.3–93.6)	6/59; 10.2% (4.7–20.5)	<0.01	15/17; 88.2% (65.7–96.7)	1/32; 3.1% (0.6–15.7)	<0.01	2/3; 66.6% (20.8–93.9)	2/13; 15.4% (4.3–42.2)	0.09	4/5; 80.0% (37.6–96.4)	3/14; 21.4% (7.6–47.6)	0.02
Ongoing pregnancy	-	7/59; 11.9% (5.9–22.5)	-	-	4/32; 12.5% (5.0–28.1)	-	-	2/13; 15.4% (4.3–42.2)	-	-	1/14; 7.1% (1.3–31.5)	-

CPR = clinical pregnancy rate; LBR = live birth rate; MR = miscarriage rate. CPR was calculated as the proportion of women (with ≥1 clinical pregnancy), whereas LBR and MR were calculated per pregnancy. Values are reported as absolute number, percentage and 95% CI. The 95% CIs were calculated using the Wilson method.

**Table 6:** Comparison of post-metroplasty obstetric outcomes between patients with partial and complete septum, and between patients with and without cervical/vaginal anomalies.

	Partial Septum (n = 38)	Complete Septum (n = 32) *	*p*	Complete Septum Without Cervical/Vaginal Anomaly (n = 16)	Complete Septum with Cervical/Vaginal Anomaly (n = 16)	*p*
CPR	32/38; 84.2% (69.6–92.6)	27/32; 84.4% (68.2–93.1)	0.51	13/16; 81.2% (57.0–93.4)	14/16; 87.5% (64.0–96.5)	0.39
LBR	27/32; 84.4% (68.2–93.1)	19/27; 70.4% (51.5–84.1)	0.14	9/13; 69.2% (42.4–87.3)	10/14; 71.4% (45.4–88.3)	1.00
MR	1/32; 3.1% (0.6–15.7)	5/27; 18.5% (8.2–36.7)	0.08	2/13; 15.4% (4.3–42.2)	3/14; 21.4% (7.6–47.6)	1.00
Ongoing pregnancy	4/32; 12.5% (5.0–28.1)	3/27; 11.1% (3.9–28.1)	1.00	2/13; 15.4% (4.3–42.2)	1/14; 7.1% (1.3–31.5)	0.60

CPR = clinical pregnancy rate; LBR = live birth rate; MR = miscarriage rate. CPR was calculated as the proportion of women (with ≥1 clinical pregnancy), whereas LBR and MR were calculated per pregnancy. * Includes both isolated complete septa (n = 16) and complete septa with associated cervical and/or vaginal anomalies (n = 16). Values are reported as absolute number, percentage and 95% CI. The 95% CIs were calculated using the Wilson method.

## Data Availability

The data presented in this study are available from the corresponding author upon request.

## Abbreviations

The following abbreviations are used in this manuscript:

3D TVUS	Three-dimensional transvaginal ultrasound
CPR	Clinical pregnancy rate
LBR	Live birth rate
MR	Miscarriage rate
ART	Assisted reproductive technology
BMI	Body mass index
EP	Ectopic pregnancy
ET	Embryo transfer
RPOC	Retained products of conception

## References

[1] Chan Y.Y., Jayaprakasan K., Zamora J., Thornton J.G., Raine-Fenning N., Coomarasamy A. (2011). The prevalence of congenital uterine anomalies in unselected and high-risk populations: A systematic review. Hum. Reprod. Update.

[2] Grimbizis G.F., Gordts S., Di Spiezio Sardo A., Brucker S., De Angelis C., Gergolet M., Li T.C., Tanos V., Brölmann H., Gianaroli L. (2013). The ESHRE/ESGE consensus on the classification of female genital tract congenital anomalies. Hum. Reprod..

[3] Noventa M., Spagnol G., Marchetti M., Saccardi C., Bonaldo G., Laganà A.S., Cavallin F., Andrisani A., Ambrosini G., Vitale S.G. (2022). Uterine Septum with or without Hysteroscopic Metroplasty: Impact on Fertility and Obstetrical Outcomes-A Systematic Review and Meta-Analysis of Observational Research. J. Clin. Med..

[4] Wang J.H., Xu K.H., Lin J., Chen X.Z. (2009). Hysteroscopic septum resection of complete septate uterus with cervical duplication, sparing the double cervix in patients with recurrent spontaneous abortions or infertility. Fertil. Steril..

[5] Chen S.Q., Deng N., Jiang H.Y., Li J.B., Lu S., Yao S.Z. (2013). Management and reproductive outcome of complete septate uterus with duplicated cervix and vaginal septum: Review of 21 cases. Arch. Gynecol. Obstet..

[6] Ludwin A., Ludwin I., Pityński K., Banas T., Jach R. (2013). Differentiating between a double cervix or cervical duplication and a complete septate uterus with longitudinal vaginal septum. Taiwan. J. Obstet. Gynecol..

[7] De A., Jain A., Tripathi R., Nigam A. (2020). Complete Uterine Septum with Cervical Duplication and Longitudinal Vaginal Septum: An Anomaly Supporting Alternative Embryological Development. J. Hum. Reprod. Sci..

[8] Miller C.M., Shenoy C.C., Khan Z. (2021). Three degrees of separation: Complete uterine and cervical septa. Fertil. Steril..

[9] Bermejo C., Martínez-Ten P., Recio M., Ruiz-López L., Díaz D., Illescas T. (2014). Three-dimensional ultrasound and magnetic resonance imaging assessment of cervix and vagina in women with uterine malformations. Ultrasound Obstet. Gynecol..

[10] Campo R., Meier R., Dhont N., Mestdagh G., Ombelet W. (2014). Implementation of hysteroscopy in an infertility clinic: The one-stop uterine diagnosis and treatment. Facts Views Vis. Obgyn..

[11] Yu L.L., Zhang X., Zhang T., Chen H.R., Wang Z.H. (2014). Detection of congenital uterine malformation by using transvaginal three-dimensional ultrasound. J. Huazhong Univ. Sci. Technol. Med. Sci..

[12] Catena U. (2022). Surgical treatment of the septate uterus. Hysteroscopy Newsl..

[13] Catena U., Bernardini F., La Fera E., Fedele C., Bonetti E., Pozzati F., Scambia G., Grimbizis G.F. (2025). Complete uterine septum, cervical septum and longitudinal vaginal septum: A challenging differential diagnosis with double cervix. Facts Views Vis. Obgyn..

[14] Fascilla F.D., Resta L., Cannone R., De Palma D., Ceci O.R., Loizzi V., Di Spiezio Sardo A., Campo R., Cicinelli E., Bettocchi S. (2020). Resectoscopic Metroplasty with Uterine Septum Excision: A Histologic Analysis of the Uterine Septum. J. Minim. Invasive Gynecol..

[15] Practice Committee of the American Society for Reproductive Medicine (2024). Evidence-based diagnosis and treatment for uterine septum: A guideline. Fertil. Steril..

[16] Pabuçcu R., Gomel V. (2004). Reproductive outcome after hysteroscopic metroplasty in women with septate uterus and otherwise unexplained infertility. Fertil. Steril..

[17] Colacurci N., De Franciscis P., Mollo A., Litta P., Perino A., Cobellis L., De Placido G. (2007). Small-diameter hysteroscopy with Versapoint versus resectoscopy with a unipolar knife for the treatment of septate uterus: A prospective randomized study. J. Minim. Invasive Gynecol..

[18] Etrusco A., Laganà A.S., Chiantera V., Gerli S., Carugno J., Sorrentino F., Riemma G., Vitagliano A., Favilli A. (2024). Efficacy, safety, and feasibility of the treatment of intrauterine pathologies with the mini-resectoscope: A systematic review. Int. J. Gynaecol. Obstet..

[19] Bifulco G., Di Spiezio Sardo A., De Rosa N., Greco E., Spinelli M., Di Carlo C., Tommaselli G.A., Nappi C. (2012). The use of an oral contraceptive containing estradiol valerate and dienogest before office operative hysteroscopy: A feasibility study. Gynecol. Endocrinol..

[20] Catena U., Bernardini F., Palermo E.B., Pozzati F., La Fera E., Moro F., Testa A.C. (2026). Standardized hysteroscopic management of complete septate uterus with duplicated cervices and longitudinal vaginal septum: A single-center experience. Int. J. Gynaecol. Obstet..

[21] Di Spiezio Sardo A., Manzi A., Zizolfi B., Giampaolino P., Carugno J., Grimbizis G. (2021). The step-by-step hysteroscopic treatment of patients with vaginal and complete uterine septum with double cervix (U2bC2V1). Fertil. Steril..

[22] Triantafyllidou O., Panagodimou E.K., Syggelos N., Vlahos N.F. (2024). Hysteroscopic treatment of complete uterine septum, double cervix and longitudinal vaginal septum (U2bC2V1): The use of a Foley catheter balloon. Facts Views Vis. Obgyn..

[23] Parsanezhad M.E., Alborzi S., Zarei A., Dehbashi S., Shirazi L.G., Rajaeefard A., Schmidt E.H. (2006). Hysteroscopic metroplasty of the complete uterine septum, duplicate cervix, and vaginal septum. Fertil. Steril..

[24] Zizolfi B., Spiezio Sardo A.D., Gallo A., Virgilio A., Fabbri M., Manzi A., Casadio P. (2025). Surgical and reproductive outcomes of hysteroscopic metroplasty for complete septate uterus with or without associated cervical anomalies and/or vaginal septa. Eur. J. Obstet. Gynecol. Reprod. Biol..

